# Characterisation of Zika virus infection in primary human astrocytes

**DOI:** 10.1186/s12868-018-0407-2

**Published:** 2018-02-20

**Authors:** Michal Stefanik, Petra Formanova, Tomas Bily, Marie Vancova, Ludek Eyer, Martin Palus, Jiri Salat, Carla Torres Braconi, Paolo M. de A. Zanotto, Ernest A. Gould, Daniel Ruzek

**Affiliations:** 1 Department of Virology, Veterinary Research Institute, Hudcova 70, 62100 Brno, Czech Republic; 2 Institute of Parasitology, Biology Centre of the Czech Academy of Sciences, Branisovska 31, 37005 Ceske Budejovice, Czech Republic; 3 Faculty of Science, University of South Bohemia, Branisovska 31, 37005 Ceske Budejovice, Czech Republic; 4 Laboratory of Molecular Evolution and Bioinformatics, Department of Microbiology, Institute of Microbiology Sciences, University of São Paulo, São Paulo, 05508-000 Brazil; 5 EHESP French School of Public Health, IRD French Institute of Research for Development, EPV UMR_D 190 Emergence des Pathologies Virales, Aix Marseille Université, Marseille, France

**Keywords:** Zika virus, Flavivirus, Astrocyte, Luminex, Immune response, Electron tomography

## Abstract

**Background:**

The recent Zika virus (ZIKV) outbreak has linked ZIKV with microcephaly and other central nervous system pathologies in humans. Astrocytes are among the first cells to respond to ZIKV infection in the brain and are also targets for virus infection. In this study, we investigated the interaction between ZIKV and primary human brain cortical astrocytes (HBCA).

**Results:**

HBCAs were highly sensitive to representatives of both Asian and African ZIKV lineages and produced high viral yields. The infection was associated with limited immune cytokine/chemokine response activation; the highest increase of expression, following infection, was seen in CXCL-10 (IP-10), interleukin-6, 8, 12, and CCL5 (RANTES). Ultrastructural changes in the ZIKV-infected HBCA were characterized by electron tomography (ET). ET reconstructions elucidated high-resolution 3D images of the proliferating and extensively rearranged endoplasmic reticulum (ER) containing viral particles and virus-induced vesicles, tightly juxtaposed to collapsed ER cisternae.

**Conclusions:**

The results confirm that human astrocytes are sensitive to ZIKV infection and could be a source of proinflammatory cytokines in the ZIKV-infected brain tissue.

## Background

Zika virus (ZIKV) is an emerging mosquito-borne member of the genus *Flavivirus* and family *Flaviviridae*. ZIKV was firstly isolated from a rhesus monkey in the Zika forest in Uganda in 1947 [1]. The virus had been endemic in Africa [2] and Asia, but since 2007 its geographic distribution has significantly increased, reaching Yap Islands (2007), French Polynesia (2013), and South America (2015) [3]. Most cases of ZIKV infections in humans are asymptomatic; approximately 20% of clinically affected people mostly experience mild symptoms, such as fever, arthralgia, myalgia, and rash [3]. The recent ZIKV outbreaks linked the ZIKV infection with rare cases of Guillain–Barré syndrome [4], and there is also evidence that ZIKV can cause devastating neonatal central nervous system (CNS) malformations, most prominently microcephaly [5], and other neurological disorders in adults (myelitis, meningoencephalitis, etc.) [6, 7], including fatal ZIKV neuroinfections [8, 9]. Astrocytes have critical roles in host defence during viral infections of the CNS, and are among the first cells to respond to ZIKV infection in the brain as well as being targets for virus infection [10, 11]. After peripheral inoculation of immunocompetent mice on the day of birth with ZIKV, the astrocytes were found to be the first cells targeted in the brain [11]. Similarly, intracerebral inoculation of newborn and 5-week-old mice with ZIKV reveals the replication of the virus in both neurons as well as astroglial cells [10]. All these observations indicate that astrocytes play a crucial role during the development of ZIKV neuroinfection. Despite the importance of astrocytes during ZIKV infection in the CNS, information concerning the interaction between ZIKV and human astrocytes remains largely limited [12]. In this study, human brain cortical astrocytes (HBCA) were used to characterize ZIKV infection in astrocytes in terms of viral growth, cytokine/chemokine and growth factor production, and 3D ultrastructural changes in infected cells.

## Methods

### Growth of ZIKV in human astrocytes

**Fig. 1:**
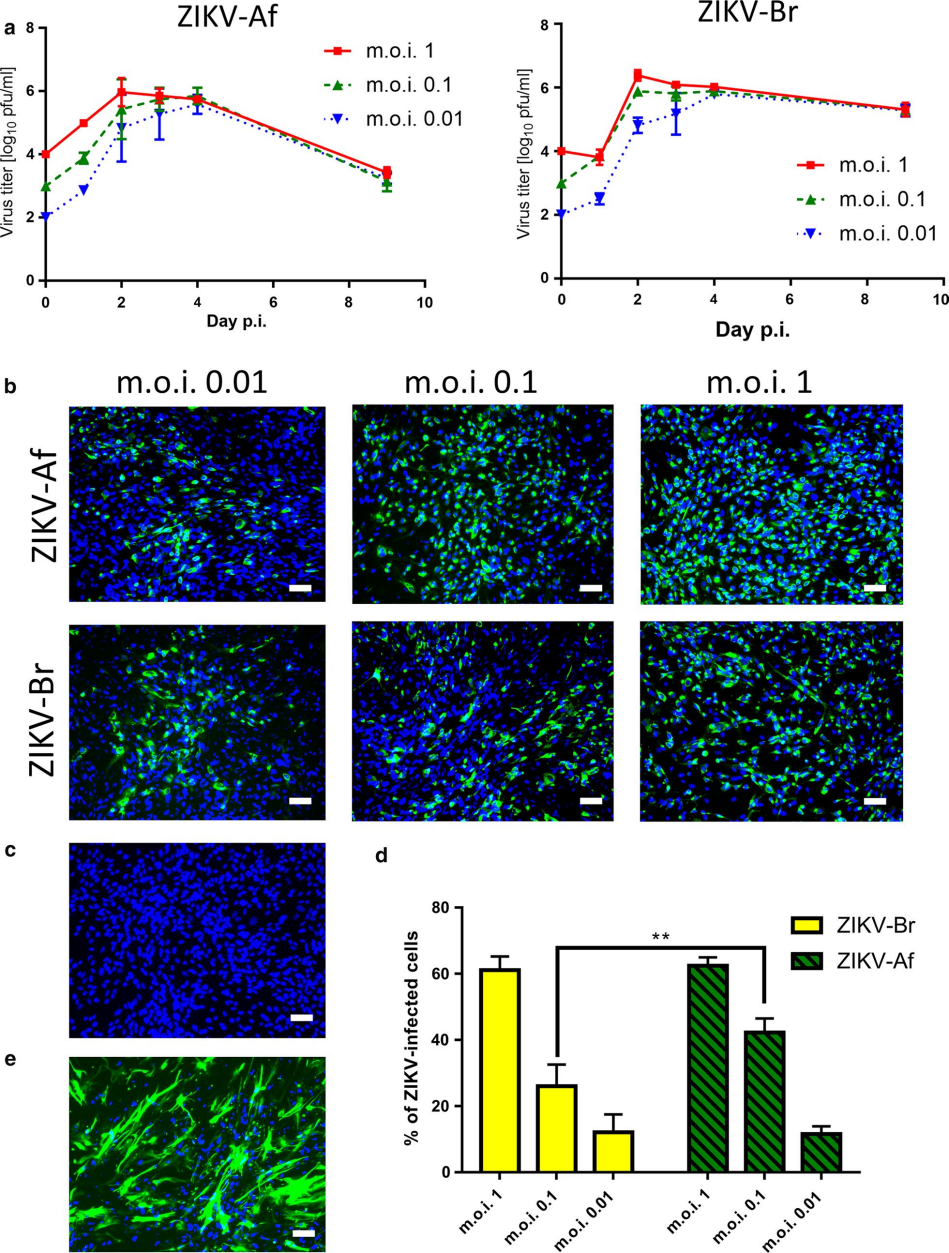
**a** Viral titres in culture supernatant from HBCAs infected with ZIKV-Af and ZIKV-Br and collected at 0, 1, 2, 3, 4 and 9 days p.i. were determined by plaque assay using Vero cells. Viral titres are expressed as p.f.u./ml. Data are from two independent experiments done in triplicates and represent mean ± SEM. **b** HBCAs infected with different doses of ZIKV-Af and ZIKV-Br were grown and fixed on slides at day 2 p.i. were stained with anti-flavivirus envelope antibody (green) and counterstained with DAPI (blue). Bar, 50 µm. **c** Mock-infected HBCAs stained with primary and secondary antibodies were used as a negative control, and did not exhibit any ZIKV antigen staining. Bar, 50 µm. **d** The percentage of HBCAs that were positive for ZIKV antigen in culture on day 2 p.i. Data were obtained based on a total of 23,000 cells per group counted in at least ten independent fields. Data were obtained from two independent experiments done in triplicates with two different batches of astrocytes, are expressed as mean ± SEM and were analysed using Two-way ANOVA (GraphPad Prism 5.04). **p < 0.01. **e** Expression of GFAP, a marker of astrocytes, was demonstrated in HBCA culture stained with anti-GFAP antibody (green) and counterstained with DAPI (blue). Bar, 50 µm

We employed a plaque assay and immunofluorescence staining for viral antigen to determine ZIKV infection and replication kinetics in adult HBCAs (purchased from ScienCell at passage 1 and propagated in Astrocyte medium (Thermo Fisher Scientific) with 6% foetal calf serum at 37 °C and 5% CO_2_ according to the recommendation of the manufacturer). The cells exhibited typical astrocyte morphology and expressed GFAP, as documented by staining with GFAP antibody conjugated to Alexa Fluor 488 (1 : 100, Santa Cruz) (Fig. 1e). Monolayer HBCA cultures grown in 96-well plates were inoculated with ZIKV at multiplicities of infection (m.o.i.) = 1, 0.1, and 0.01. Two ZIKV strains were used in the study, i.e., MR766, a representative of the African ZIKV lineage (further referred as ZIKV-Af) isolated in 1947 in Uganda and provided by the European Virus Archive, and ZIKV-Br, a representative of the Asian ZIKV lineage, isolated from a febrile case in the State of Paraiba in the Northeast of Brazil by the Evandro Chagas Institute (IEC) in Belém do Para, Brazil [13]. The virus was isolated and propagated in C6/36 and Vero B6 cells. At 0, 1, 2, 3, 4 and 9 days p.i., supernatant medium from appropriate wells was collected and virus titres were determined by plaque assay on Vero cells as described previously [14]. The cells were subjected to cold acetone:methanol (1:1) fixation and labelled for the presence of viral E protein using flavivirus-specific monoclonal antibody (clone D1-4G2-4-15; 1:250; Sigma-Aldrich) as described previously [15].

### Cytokine/chemokine/growth factor production by the ZIKV-infected astrocytes

To characterize the effect of ZIKV-infection on the production of cytokines/chemokines and growth factors by HBCA, the cells were infected with ZIKV-Af and ZIKV-Br at an m.o.i. of 0.1 and cell culture supernatants were collected in triplicates on days 1, 2 and 4 p.i. The concentrations of 30 cytokines, chemokines, and growth factors were measured in the cell culture media using the Human Cytokine Magnetic 30-Plex Panel for the Luminex platform (Life Technologies, Frederick, MD) and a MAGPIX instrument (Luminex, Austin, TX). All procedures were performed according to the manufacturer’s instructions and as described previously [16]. The immunoassay detects interleukin (IL)-1β, IL-1 receptor antagonist (IL-1RA), IL-2, IL-2 receptor (IL-2R), IL-4, IL-5, IL-6, IL-7, IL-8, IL-10, IL-12 (p40/p70), IL-13, IL-15, IL-17, tumour necrosis factor alpha (TNF-α), interferon alpha (IFN-α), interferon gamma (INF-γ), granulocyte colony stimulating factor (G-CSF), granulocyte macrophage colony stimulating factor (GM-CSF), eotaxin (CCL11), inducible protein (IP)-10 (CXCL10), macrophage chemotactic protein (MCP)-1, monokine induced by gamma interferon (MIG; CXCL9), macrophage inflammatory protein (MIP)-1α (CCL3), MIP-1β (CCL4), regulated upon expression normal T-cell expressed and secreted (RANTES; CCL5), and the growth factors epidermal growth factor (EGF), basic fibroblast growth factor, hepatocyte growth factor (HGF), and vascular endothelial growth factor (VEGF) [16]. Controls comprised uninfected cells and mock-infected cells with UV-inactivated virus.

### Electron tomography of the ZIKV-infected astrocytes

Morphological changes in the ZIKV-infected HBCA were investigated at a high magnification using transmission electron microscopy/electron tomography. Cells were cultured on 3 mm Sapphire discs and infected with ZIKV-Af for 48 h. Both infected and mock-infected cells a fixed in 2.5% glutaraldehyde in 0.1 H HEPES for 1 h at room temperature. After washing in buffer, disks were frozen in the presence of 20% of BSA using the EM PACT2 (Leica Microsystems, Vienna, Austria). Freeze substitution was performed in 2% OsO4 in acetone at − 90 °C for 96 h. Then the temperature was raised to − 20 °C (6 °C/h) and, after 10 h, to 4 °C. Sapphire discs were washed in acetone (15 min, three times) and infiltrated with resin EMbed 812 (EMS):acetone mixtures at 1:2, 1:1, 2:1, and finally 100% resin, for 1 h each. Discs with the cells facing down were transferred onto the surface of polymerized pure resin blocks and cured for 48 h at 60 °C. Ultrathin sections of 100 nm were placed on the formvar-carbon coated Cu grids, contrasted in ethanolic uranyl acetate (30 min) and lead citrate (20 min) and, finally, carbon coated. Ten nm gold NPs were used as fiducial markers. Images were acquired with a 200 kV JEOL 2100F transmission electron microscope equipped with a high-tilt stage and a Gatan camera (Orius SC 1000) and controlled with SerialEM automated acquisition software45. Analysis by dual axis electron tomogram was performed as a montage 2 × 2, with tilt angle ± 60° with an increment 1°, pixel resolution of tomogram 0.8 nm. Staining was performed with alcoholic uranyl acetate for 30 min, followed by lead citrate for 20 min. Electron tomograms were reconstructed using the IMOD software package. Manual masking of the area of interest was employed to generate 3D surface models.

### Statistics

Data are expressed as mean ± SEM, and the significance of differences between groups was evaluated by two-way ANOVA test, Tukey’s multiple comparison test for cytokine/chemokine production and Sidak’s multiple comparison test for ZIKV antigen positive cells using GraphPad Prism 7 (GraphPad Software, Inc., USA), version 7.04. Differences with *p* < 0.05 were considered significant.

## Results

### Growth of ZIKV in human astrocytes

Active and productive ZIKV replication of both strains investigated in the form of release of virions was first detected on days 1 and 2 p.i. Day 2 p.i. also represented a peak of virus production (Fig. 1a). The highest increase in virus production was seen with an m.o.i. of 0.01. From day 2 until day 4, there was a stable viral titre reaching approximately 6 log_10_ pfu/ml in the culture medium; a similar maximal titre was reached regardless of the m.o.i. used. On day 9 p.i., viral titre in the culture infected with ZIKV-Br was about 100-times higher when compared to ZIKV-Af. Immunofluorescence staining revealed high infection rates, reaching almost 60% of HBCA infected with both ZIKV strains at an m.o.i. of 1 on day 2 p.i. (Fig. 1b, d). Infection rates in HBCA cultures infected with m.o.i. = 0.1 of ZIKV-Br were significantly lower when compared with the infection rate of ZIKV-Af (*p* < 0.01; F_1,12_ = 7.307; t = 4.446; DF = 12; Fig. 1d).

### Cytokine/chemokine/growth factor production by the ZIKV-infected astrocytes

**Fig. 2:**
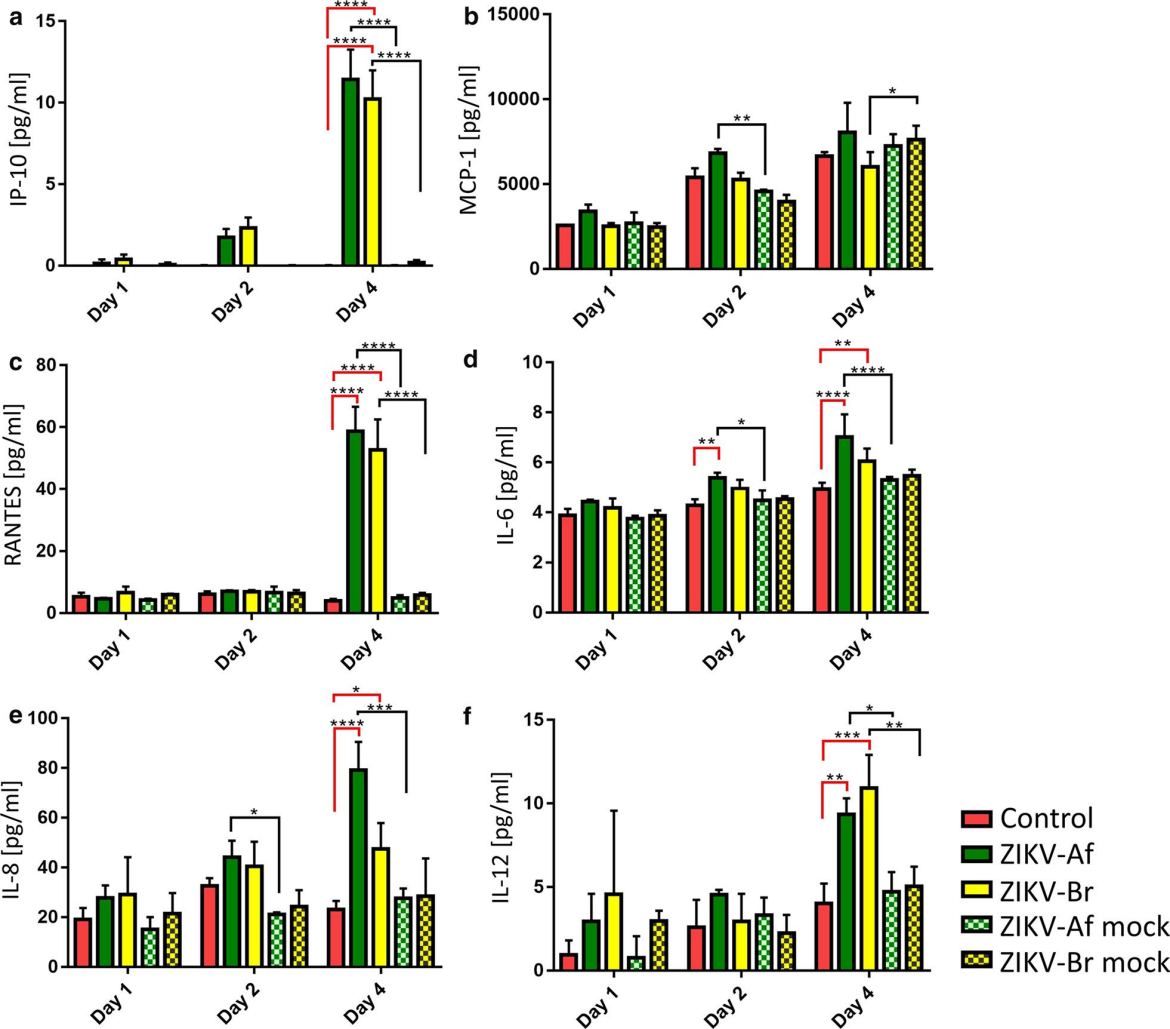
ZIKV induces the production of cytokines/chemokines in infected HBCAs. Levels of 30 cytokines, chemokines and growth factors in supernatants from ZIKV-infected HBCAs were measured using the Cytokine Human Magnetic 30-Plex Panel for the Luminex platform at 1, 2, and 4 days p.i. Uninfected or mock-infected HBCAs (infected with UV-inactivated virus) were used as controls. Significant differences in the cytokine/chemokine production were observed in case of CXCL-10/IP-10 (**a**), CCL2/MCP-1 (**b**), CCL5/RANTES (**c**), IL-6 (**d**), IL-8 (**e**), and IL-12 (**f**). Data were obtained after measurements from three replicates and are expressed as mean ± SEM and were analysed using two-way ANOVA (GraphPad Prism 5.04). *p < 0.05; **p < 0.01; ***p < 0.001; ****p < 0.0001

To characterize the effect of ZIKV-infection on the production of cytokines/chemokines and growth factors by HBCA, the cells were infected with ZIKV-Af and ZIKV-Br, and the concentrations of 30 cytokines, chemokines, and growth factors were measured in the cell culture media using the Human Cytokine Magnetic 30-Plex Panel for the Luminex platform. No significant changes in the production of IL-1β, IL-2R, IL-4, IL-5, IL-7, IL-10, IL-13, IL-15, IL-17, TNF-α, IFN-α, IFN-γ, G-CSF, GM-CSF, eotaxin, MIG, MIP-1α, MIP-1β, EGF, HGF, and VEGF were seen in the ZIKV-infected astrocytes when compared with mock-treated controls at any time point investigated (*p* > 0.05; data not shown). A slightly increased concentration of MCP-1 was seen in cultures infected with ZIKV-Af on day 2 p.i. (*p* < 0.01; F_4,10_ = 9.586; TukeyQ = 5.963; DF = 30). On the other hand, the concentration of MCP-1 in cultures infected with ZIKV-Br was lower (*p* < 0.05; F_4,10_ = 9.586; TukeyQ = 4.296; DF = 30) in comparison with mock-infected controls on day 4 (Fig. 2b). On day 4 p.i., there was significantly increased production of IP-10 (*p* < 0.0001; F_4,10_ = 81.85; TukeyQ = 24.95; DF = 30 for ZIKV-Br, F_4,10_ = 81.85, TukeyQ = 28.34, DF = 30 for ZIKV-Af, when compared to mock-infected cells), RANTES (*p* < 0.0001; F_4,10_ = 59.02, TukeyQ = 23.90, DF = 30 for ZIKV-Br; F_4,10_ = 59.02, TukeyQ = 27.38, DF = 30 for ZIKV-Af when compared to mock-infected cells), and IL-12 (ZIKV-Af, *p* < 0.05; F_4,10_ = 8.593, TukeyQ = 4.512, DF = 30; ZIKV-Br, *p* < 0.01, F_4,10_ = 8.593, TukeyQ = 5.729, DF = 30, when compared to mock-infected cells) in astrocytes infected with either of the ZIKV strains in comparison with the mock-infected and uninfected controls (Fig. 2). Concentration of IL-6 and IL-8 was increased only in cultures infected with ZIKV-Af on days 2 (IL-6, *p* < 0.05, F_4,10_ = 14.03, TukeyQ = 4.468, DF = 30; IL-8, *p* < 0.05, F_4,10_ = 19.85, TukeyQ = 4.736, DF = 30) and 4 (IL-6, *p* < 0.0001, F_4,10_ = 14.03, TukeyQ = 8.408, DF = 30; IL-8, *p* < 0.001, F_4,10_ = 19.85, TukeyQ = 10.62, DF = 30) when compared to cultures mock-infected with UV-inactivated viruses (Fig. 2d, e).

### Electron tomography of the ZIKV-infected astrocytes

**Fig. 3:**
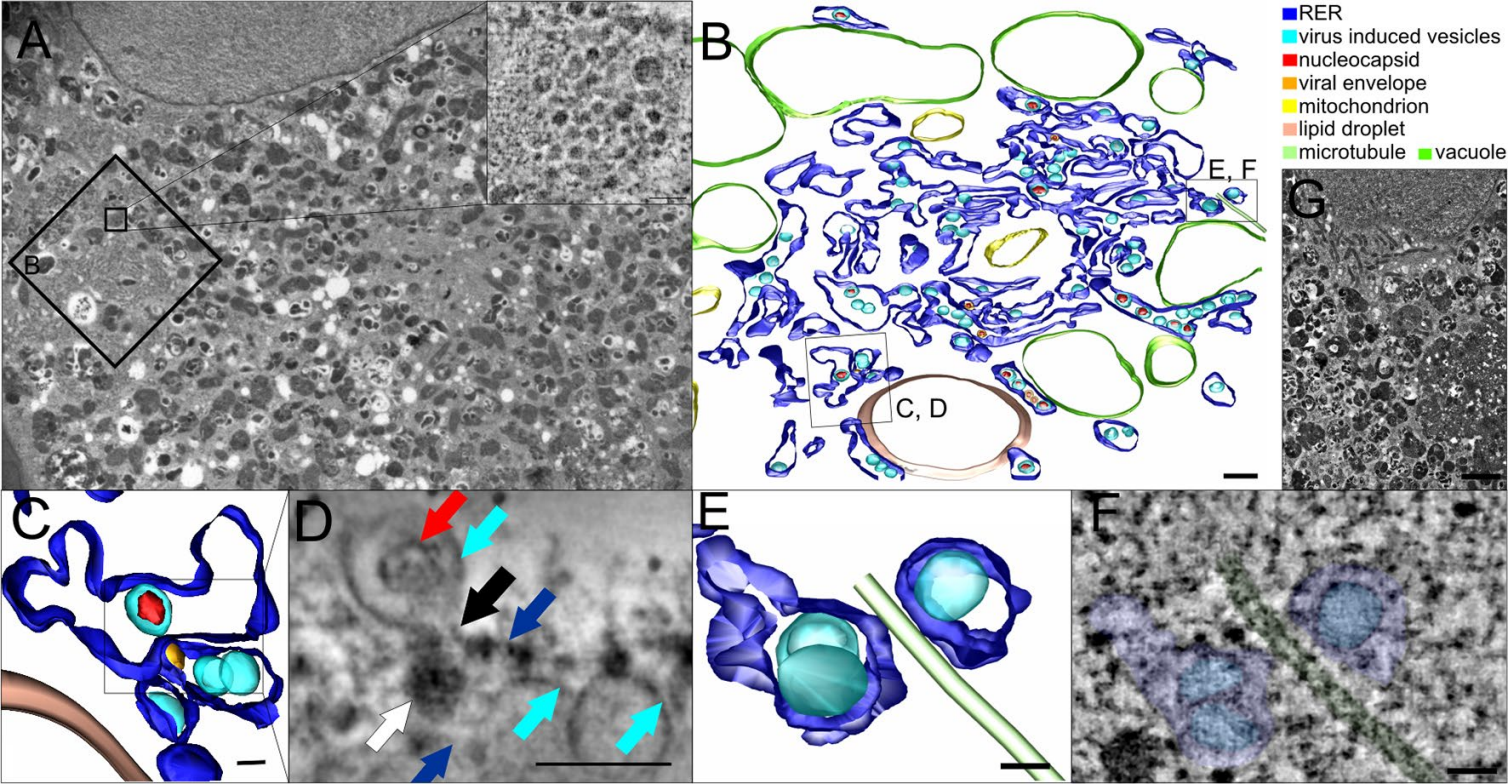
Replication of ZIKV in astrocytes, 48 h p.i. **A** The cytoplasm filled with numerous vacuoles containing neurosecretory vesicles (details in insets). **B** 3D model of the replication site. **C**, **D** Putative budding event—virus-induced vesicle with the nucleocapsid is connected (black arrow) to the neighbouring cisternae of the rough endoplasmic reticulum. Newly formed virion (white arrow) is located below the channel. **E**, **F** Microtubule associated to the two cisternae of the rough endoplasmic reticulum. **G** Cytoplasm of uninfected cells was filled with numerous vesicles. Bars: 2 µm (**A**, **G**), 200 nm (**B**), 100 nm (**A**-inset, **D**), and 50 nm (**C**, **E**, **F**)

Morphological changes in the ZIKV-infected HBCA were investigated using electron tomography at 48 h p.i. The astrocytes contained multiple large vacuoles in their cytoplasm packed with neurosecretory vesicles of various sizes (50.9 ± 12.2 nm in diameter; n = 15) (Fig. 3A inset); however, these vacuoles were not associated with the infection and were found also in control cells (Fig. 3G). ZIKV virions (50.6 ± 1.9 nm in outer dimeter; n = 6) were found in perinuclear replication sites within extensively rearranged endoplasmic reticulum (ER) cisternae (Fig. 3B). ZIKV-induced ER sub-compartments were spherical (85.8 ± 8.3 nm, n = 70), and some of them contained viral nucleocapsids.

We also observed a tightly juxtaposed ER cisternae and the channel/openings of the virus-induced vesicles oriented to the second closely associated ER cisternae where the round structure (the spherule with the outer diameter of 58.8 nm) was present (Fig. 3C, D).

There were connections between cellular microtubules and vacuoles that harboured the virus induced vesicles (Fig. 3E, F). Furthermore, microtubules were found associated with the convoluted membranes.

## Discussion

Astrocytes are targets for ZIKV infection in the brain and may play a crucial role in the development of ZIKV-associated neurological complications observed in humans. Despite the importance of astrocytes during ZIKV infection in the CNS, information concerning the interaction between ZIKV and human astrocytes remains largely limited. This study investigated the interaction of ZIKV with primary human cortical astrocytes in terms of ZIKV growth and cytokine/chemokine/growth factor production by the ZIKV-infected astrocytes. We also used 3D electron tomography to characterize ultrastructural changes associated with ZIKV-infection in human astrocytes. We found that human cortical astrocytes are sensitive to representatives of both ZIKV lineages. At early time points, replication kinetics and viral yields were similar for both ZIKV strains; however, ZIKV-Af exhibited higher infection rate on day 2 if m.o.i. 0.1 was used. Similarly to our study, Hamel et al. [17] observed comparable replication kinetics and viral yields for human astrocytes infected with two clinical ZIKV isolates representing both ZIKV lineages (H/PF/2013 and HD 78788). In contrast, Simonin et al. [12] reported a higher virus production if the astrocytes were infected with African ZIKV strain (ArB41644) when compared with an Asian ZIKV representative (strain H/PF/2013). Taken together these results indicate that the sensitivity of human astrocytes is more likely strain-dependent than ZIKV lineage dependent.

Production of 30 cytokines, chemokines, and growth factors by the ZIKV-infected astrocytes was measured in culture media at 1, 2, and 4 days p.i. The analysis showed that the infection was associated with limited immune cytokine/chemokine response activation; the highest increase of expression, following infection, was seen in CXCL-10 (IP-10), interleukin (IL)-6, 8, 12, and CCL5 (RANTES). This indicates that ZIKV infection of the HBCA is associated with an increased expression of a relatively narrow spectrum of proinflammatory cytokines and chemokines. When compared with cytokine/chemokine expression by HBCA infected with the flavivirus, tick-borne encephalitis virus (TBEV) [15], the induction of expression of the cytokines/chemokines by the ZIKV-infected HBCAs was substantially lower. These results are consistent with a study by Hanners et al. [18], who reported that ZIKV stimulates a poor immune response in human neuroprecursors. Relatively limited activation of the immune response in ZIKV-infected HBCAs may be associated with high infection rates and high titre virus production. Hamel et al. [17] performed a transcriptomic analysis in astrocytes and observed increased expression of various cytokines/chemokines at the mRNA level following infection with representatives of Asian and African ZIKV lineages, albeit the kinetics of the expression between these two lineages was different. Our study confirms the activation of expression of a limited number of cytokines/chemokines in ZIKV-infected astrocytes at a protein level. ZIKV-Af activated production of more cytokines than ZIKV-Br, since IL-6 and IL-8 were increasingly produced by astrocytes infected with ZIKV-Af but not ZIKV-Br when compared to the production by cells mock-infected with UV-inactivated virus. These results also show that astrocytes can be an important source of proinflammatory cytokines and chemokines in the ZIKV-infected human brain.

Electron tomography analysis of the ZIKV-infected astrocytes revealed extensively rearranged and proliferated ER with numerous spherical sub-compartments (85.8 ± 8.3 nm, n = 70). Some of the ER-derived sub-compartments contained viral nucleocapsids. These observation are in accordance with those of Offerdahl et al. [19] (ZIKV-induced vesicular replication compartments, 60–100 nm) and Cortese et al. [20] (ZIKV-induced vesicles had 80.82 ± 0.96 nm (n = 243) for the MR766 strain and 88.31 (± 1.25 nm; n = 219) for the H/PF/2013 strain, respectively. In contrast to Offerdahl et al. [19], we did not detect hollow shaped spheres of 20–30 nm in diameter inside virus-induced vesicles nor tubular replication compartments. We noticed differences in measurements of outer diameters of ZIKV measured by means of TEM on ultrathin sections that were probably caused by different EM specimen preparation methods (40 nm [20]; ~ 30 nm [19]; 47 ± 3 nm, n = 13, [21]).

Recently, Rossignol et al. [21] and Cortese et al. [20] described the presence of a pore opening in ZIKV-induced spherules at the cytoplasmic side of the ER and the presence of viruses in apposed ER cisternae. Similar structures, i.e., tightly juxtaposed ER cisternae and the channel/openings of the virus-induced vesicles oriented to the second closely associated ER cisternae, were found also in our study (Fig. 3C, D). Our observation supports and extends the hypothesis that the site of viral budding into the ER is closely apposed to the other ER cisternae where the site of viral RNA replication is located (inside the virus-induced vesicle) [20–22] and both ER sub-compartments are connected by a channel. There were connections between cellular microtubules and vacuoles that harboured the virus induced vesicles in ZIKV-infected HBCAs, which is in accordance with other reports when HBCAs were infected with another flavivirus, TBEV [15, 23]. Monel et al. [24] described the presence of large cytoplasmic vacuoles derived from the endoplasmic reticulum in both primary skin fibroblasts and human astrocytes that were infected (24 h p.i.) with ZIKV HD78 (m.o.i. of 1). However, in this study the presence of multiple large vacuoles in the cytoplasm packed with neurosecretory vesicles of various sizes was found also in control (uninfected) HBCAs, so their presence in astrocytes seems to be not associated with the infection.

## Conclusions

Our results show that HBCAs are sensitive to representatives of African and Asian ZIKV lineages. We detected a high infection rate in HBCAs infected with ZIKV and the cells produced high virus titres. Infection of HBCAs is associated with activation of production of a limited spectrum of cytokines and chemokines. ZIKV-infected HBCAs undergo extensive cellular remodelling at an ultrastructural level, mainly manifested as a proliferation and reorganization of ER. The results also indicate that astrocytes are important targets for ZIKV and may be a source of proinflammatory cytokines and chemokines in the infected brain. Our observations are in good accordance with other studies suggesting that African ZIKV strains are capable of causing similar damage to CNS cells as the strains from Asian ZIKV lineage [12, 25]. In addition, recent reports indicated that both African and Asian ZIKV lineages appear to be capable of causing microcephaly in children [25].

## Abbreviations

BSA: bovine serum albumin
CCL11: eotaxin
DF: degrees of freedom
EGF: epidermal growth factor
ER: endoplasmic reticulum
ET: electron tomography
G-CSF: granulocyte colony stimulating factor
GFAP: glial fibrillary acidic protein
GM-CSF: granulocyte macrophage colony stimulating factor
HBCA: human brain cortical astrocytes
HGF: hepatocyte growth factor
IFN-α: interferon alpha
INF-γ: interferon gamma
IL: interleukin
IP-10: inducible protein 10 (CXCL10)
MCP-1: macrophage chemotactic protein
MIG: monokine induced by gamma interferon (CXCL9)
MIP: macrophage inflammatory protein
m.o.i.: multiplicity of infection
pfu: plaque forming unit
RANTES: regulated upon expression normal T cell expressed and secreted (CCL5)
RER: rough endoplasmic reticulum
TBEV: tick-borne encephalitis virus
TNF-α: tumour necrosis factor alpha
VEGF: vascular endothelial growth factor
ZIKV: Zika virus
